# Supplementary material for: Dynamic transcriptomic profiles of zebrafish gills in response to zinc supplementation
Source: BMC Genomics. 2010 Oct 11;11:553. doi: 10.1186/1471-2164-11-553 (PMC3091702; doi:10.1186/1471-2164-11-553)
Supplement: Additional file 2 — Interactive Direct Interaction Network representing the molecular interactions between zinc, copper, iron, calcium and proteins encoded by transcripts changed by zinc supplementation. Mini web-site containing index.html and hyperlinked pages in subdirectory describing a Direct Interaction Network automatically generated based on curated interactions contained within the proprietary PathwayArchitect database. Ovals represent proteins and the circles symbolize metal ions. Objects are coloured by their abundance in zebrafish at the time-point they were significantly different from the control is a scale from -4 fold (dark green) to +4 fold (dark red). Where significant differences were found at more than one time-point, the colour overlay shows expression at the first instance. Dark blue squares denote 'binding', and light blue squares 'expression'; green squares stand for 'regulation', green diamonds for 'metabolism', and green circles for 'promoter binding'. Arrow heads indicate directionality of the interaction where annotated. All nodes and edges can be further interrogated by selecting the relative area of the image. [file 1471-2164-11-553-S2.zip › PathwayArchitect Zn xs DIN/83773.html]

# PROTEIN: NR1I2

|  |  |
| --- | --- |
| Name | NR1I2 |
| Type | PROTEIN |
| Description | nuclear receptor subfamily 1, group I, member 2 |
| Note | The gene product belongs to the nuclear receptor superfamily, members of which are transcription factors characterized by a ligand-binding domain and a DNA-binding domain. The encoded protein is a transcriptional regulator of the cytochrome P450 gene CYP3A4, binding to the response element of the CYP3A4 promoter as a heterodimer with the 9-cis retinoic acid receptor RXR. It is activated by a range of compounds that induce CYP3A4, including dexamethasone and rifampicin. The gene product contains a zinc finger domain. Three alternatively spliced transcripts that encode different isoforms have been described, one of which encodes two products through the use of alternative translation initiation codons. Additional transcript variants derived from alternative promoter usage, alternative splicing, and/or alternative polyadenylation exist, but they have not been fully described. |
| Alias | nuclear receptor subfamily 1 group 1 member 2 |
|  | MGC108643 |
|  | PAR |
|  | NR1I2 |
|  | PRR |
|  | pregnane X receptor |
|  | Steroid and xenobiotic receptor |
|  | Pxr |
|  | PXR.2 |
|  | nuclear receptor subfamily 1, group 1, member 2 |
|  | SAR |
|  | PAR1 |
|  | ONR1 |
|  | Orphan nuclear receptor PAR1 |
|  | PXR1 |
|  | BXR |
|  | SXR |
|  | PXR.1 |
|  | PXR |
|  | PAR2 |
|  | Pregnane X receptor |
|  | mPXR |
|  | steroid and xenobiotic receptor |
|  | PARq |
|  | pregnane X receptor (nuclear receptor sub family 1 group I member 2) |
|  | Nr1i2 |


---

|  |  |
| --- | --- |
| GO Component | nucleus |


---

|  |  |
| --- | --- |
| GO ID | GO:0003707 |
|  | GO:0003677 |
|  | GO:0006355 |
|  | GO:0008202 |
|  | GO:0005515 |
|  | GO:0003700 |
|  | GO:0005634 |
|  | GO:0007165 |
|  | GO:0004872 |
|  | GO:0006805 |
|  | GO:0004879 |
|  | GO:0046872 |
|  | GO:0016481 |
|  | GO:0003713 |
|  | GO:0006350 |
|  | GO:0016564 |


---

|  |  |
| --- | --- |
| MIM | MIM:603065 |


---

|  |  |
| --- | --- |
| Connectivity | 436 |


---

|  |  |
| --- | --- |
| Entrez ID | 18171 |
|  | 8856 |
|  | 84385 |


---

|  |  |
| --- | --- |
| Agilent ID | A\_23\_P109788 |
|  | A\_14\_P132184 |
|  | A\_53\_P141270 |
|  | A\_43\_P12749 |
|  | A\_51\_P266958 |
|  | A\_53\_P128644 |
|  | A\_23\_P109785 |


---

|  |  |
| --- | --- |
| Cellular Localization | Nucleus |
|  | Organelle |
|  | Cell |


---

|  |  |
| --- | --- |
| Pathway | Zn xs inventory |
|  | Zn xs DIN |


---

|  |  |
| --- | --- |
| GO Process | signal transduction |
|  | negative regulation of transcription |
|  | regulation of transcription, DNA-dependent |
|  | xenobiotic metabolism |
|  | transcription |
|  | steroid metabolism |


---

|  |  |
| --- | --- |
| UniGene | Rn.48713 |
|  | Hs.7303 |
|  | Mm.8509 |


---

|  |  |
| --- | --- |
| Affymetrix Probeset ID | 1369270\_at |
|  | 140371\_at |
|  | 1425723\_at |
|  | 1451807\_at |
|  | 207202\_s\_at |
|  | 207203\_s\_at |
|  | 240035\_at |
|  | 35114\_at |
|  | 1398705\_at |
|  | 53775\_at |
|  | 70745\_at |
|  | 93696\_at |
|  | g11863133\_3p\_a\_at |
|  | g3511137\_3p\_a\_at |
|  | Hs.7303.0.A1\_3p\_at |
|  | Hs.7303.0.A1\_3p\_x\_at |
|  | Msa.32374.0\_s\_at |
|  | RC\_T23830\_at |
|  | TC38246\_at |


---

|  |  |
| --- | --- |
| GO Function | ligand-dependent nuclear receptor activity |
|  | transcriptional repressor activity |
|  | DNA binding |
|  | protein binding |
|  | transcription coactivator activity |
|  | transcription factor activity |
|  | steroid hormone receptor activity |
|  | receptor activity |
|  | metal ion binding |


---

|  |  |
| --- | --- |
| Nucleotide | NM\_010936 |
|  | BC091138 |
|  | NM\_003889 |
|  | AJ009937 |
|  | AF031814 |
|  | AF151377 |
|  | AF364606 |
|  | AF061056 |
|  | NM\_033013 |
|  | NM\_052980 |
|  | AF084644 |
|  | AK122990 |
|  | AK011108 |
|  | NM\_022002 |
|  | AY007189 |
|  | AK018630 |
|  | AJ009936 |
|  | AK223456 |
|  | BC017304 |
|  | AF084645 |
|  | AY091855 |


---

|  |  |
| --- | --- |
| Protein | AAH17304 |
|  | NP\_035066 |
|  | AAC39964 |
|  | NP\_148934 |
|  | CAB55494 |
|  | BAB27404 |
|  | AAK38722 |
|  | O75469 |
|  | CAB55489 |
|  | AAC64558 |
|  | AAD47214 |
|  | BAD97176 |
|  | NP\_071285 |
|  | AAK38721 |
|  | O54915 |
|  | CAB55490 |
|  | CAB55493 |
|  | AAM26736 |
|  | AAC64557 |
|  | AAK38720 |
|  | Q9R1A7 |
|  | AAD05436 |
|  | NP\_443212 |
|  | NP\_003880 |
|  | CAB55491 |
|  | BAB31316 |
|  | CAB55492 |
|  | AAH91138 |
|  | AAG23345 |


---

|  |  |
| --- | --- |
| Organism | Mammal |


---

|  |  |
| --- | --- |
| Location | chromosome 3, 3q12-q13.3 (Homo sapiens) |
|  | chromosome 11, 11q21 (Rattus norvegicus) |
|  | chromosome 16, 16 B3 (Mus musculus) |


---

|  |  |
| --- | --- |
